# A quantitative analysis of the use of anonymization in biomedical research

**DOI:** 10.1038/s41746-025-01644-9

**Published:** 2025-05-14

**Authors:** Thierry Meurers, Karen Otte, Hammam Abu Attieh, Farah Briki, Jérémie Despraz, Mehmed Halilovic, Bayrem Kaabachi, Vladimir Milicevic, Armin Müller, Grigorios Papapostolou, Felix Nikolaus Wirth, Jean Louis Raisaro, Fabian Prasser

**Affiliations:** 1 Health Data Science Center, Berlin Institute of Health at Charité – Universitätsmedizin Berlin, Berlin, Germany; 2 Biomedical Data Science Center, Centre Hospitalier Universitaire Vaudois, Lausanne, Switzerland

**Keywords:** Translational research, Scientific data, Data publication and archiving

## Abstract

Anonymized biomedical data sharing faces several challenges. This systematic review analyzes 1084 PubMed-indexed studies (2018–2022) using anonymized biomedical data to quantify usage trends across geographic, regulatory, and cultural regions to identify effective approaches and inform implementation agendas. We identified a significant yearly increase in such studies with a slope of 2.16 articles per 100,000 when normalized against the total number of PubMed-indexed articles (*p* = 0.021). Most studies used data from the US, UK, and Australia (78.2%). This trend remained when normalized by country-specific research output. Cross-border sharing was rare (10.5% of studies). We identified twelve common data sources, primarily in the US (seven) and UK (three), including commercial (seven) and public entities (five). The prevalence of anonymization in the US, UK, and Australia suggests their practices could guide broader adoption. Rare cross-border anonymized data sharing and differences between countries with comparable regulations underscore the need for global standards.

## Introduction

Digital technology is transforming healthcare delivery, improving patient outcomes, and facilitating more efficient public health strategies^1^. Data plays a central role in these advancements and is the foundation for personalized medicine^2,3^, epidemiological research^4,5^, and the development of artificial intelligence (AI) and machine learning models in healthcare^6^. Realizing the full potential of these developments requires the ability to re-use and share health data across systems, disciplines, and borders^7^. Such data flows face significant ethical, legal, and societal challenges which remain a major roadblock, as highlighted also during the COVID-19 pandemic^8^. While obtaining informed consent, i.e., having patients opt-in to their data being shared, is a common solution, this process is faced with several challenges, including impracticality^9^ and varying levels of comprehension^10^. As an alternative, and generally as part of a privacy-by-design approach to medical research, technology can be employed to ensure that patients remain anonymous. In this context, the scientific community is working on a wide range of technologies, including federated learning^11,12^, distributed data analytics platforms^13^, differential privacy^14^, and synthetic data generation^15^. However, these techniques have not yet been adopted on a broad scale in biomedical research due to their high infrastructure requirements, complexity of use, and potential impacts on the reliability of results^16,17^. The traditional solution is data anonymization.

Anonymization, in essence, denotes a technical process of altering data in such a manner that the risk of it being traced back to individuals is significantly reduced^18^. Common methods include the removal of data, the masking of values, the addition of noise or the reduction of the fidelity of data points. The feasibility of achieving privacy protection with such methods while ensuring that the data remains useful for scientific research is a subject of intense debate within scholarly discourse^19^. Some experts argue that privacy risks are not sufficiently mitigated by this approach, suggesting a potential inadequacy in legal frameworks^20,21^. Studies that focus on the uniqueness of data as a proxy for privacy risks support this view^22,23^. Conversely, others argue that the chances of privacy breaches to actually happen are low, especially when anonymization adheres to best practices and established policies^24–26^. A range of such best practices and case studies have been published^27–30^.

Diverse perspectives on anonymization in medical research also appear across various legal frameworks. For example, in the United States (US) biomedical data is frequently shared in a “de-identified” form (see Section “Search strategy and selection criteria”) for research purposes based on the concrete requirements laid out in the Safe Harbor method of the Privacy Rule of the Health Insurance Portability and Accountability Act (HIPAA)^31^. As another example, the National Health Services (NHS) in the United Kingdom (UK) has provided specific guidance regarding the use of statistical anonymization methods^32^. In contrast, in the European Union (EU) anonymization is more challenging to apply in practice, due to ambiguities in the legal definition^33^ and a lack of a common understanding and standards^34^. To address these obstacles and facilitate data sharing, the European Union (EU) is currently introducing legislation on the so-called European Health Data Space (EHDS), which will provide additional legal bases for sharing biomedical data on an opt-out basis^35^.

The objective of this paper is to investigate, on a quantitative level, how diverse perceptions and legal ambiguities actually impact the use of anonymization in biomedical research. To this end, we analyzed a corpus of medical studies explicitly stating in their title or abstract that they used anonymized data. With our results, we aim to support policymakers, healthcare organizations, and researchers with data for improving existing policies and structures as well as developing future research agendas. Specifically, we aim to provide insights along three dimensions:

1. **Temporal trends:** Is the number of studies based on anonymized data increasing or decreasing? Did recent developments, such as the COVID-19 pandemic, have measurable impacts on the use of anonymization in biomedical research?
2. **Geographical differences:** In which countries is the sharing of anonymized data more prevalent than in others? Do frequently discussed regulatory and cultural differences across regions influence the use of anonymization in biomedical research, including in cross-border settings?
3. **Common data sources:** Are there specific organizations that frequently share data, and do these organizations target research on particular disease categories?

To the best of our knowledge, this is the first review that adopts a bottom-up approach by analyzing scientific studies that utilized anonymized data, rather than analyzing policies or anonymization practices.

## Results

**Fig. 1:**
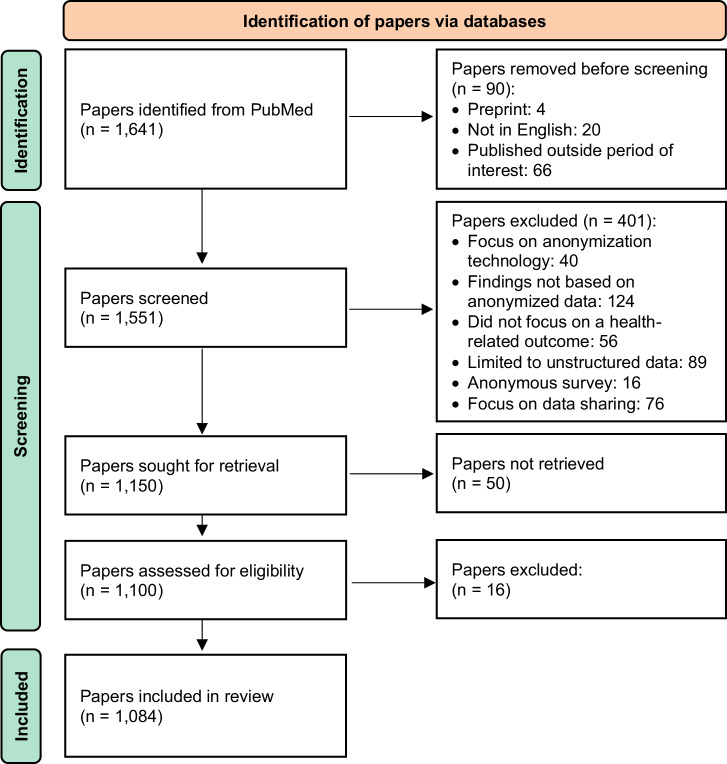
PRISMA flowchart for the search and selection process. The flowchart outlines the number of papers included and excluded in each stage of the review process.

Through the PubMed search, 1641 articles were identified. After filtering for language, peer-review status, and publication date, 1551 articles were initially screened based on their title and abstract. Of the remaining 1150 articles, 50 were inaccessible, and 16 were further excluded during full-text screening. Ultimately, 1084 articles were included in the review. The detailed selection process is depicted in the flowchart in Fig. 1.

### Temporal trends

**Fig. 2:**
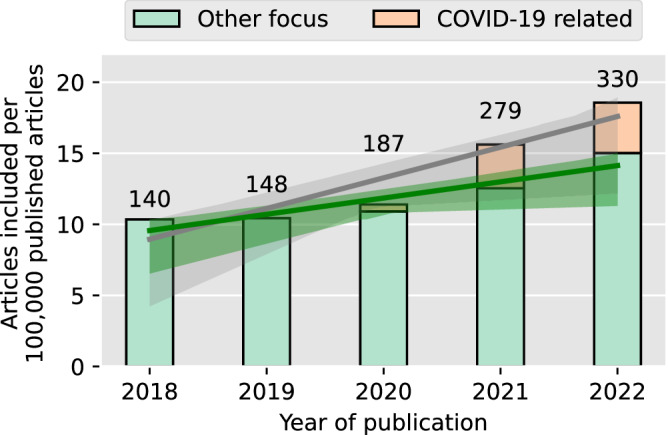
Number of articles included annually. The number of articles was normalized per 100,000 articles registered on PubMed in each year. Articles related to COVID-19 research are highlighted. Two regression lines with a 95% confidence interval, shown as two shaded areas, depict trends for the total number of articles (gray) and the number of articles without COVID-19 focus (green). Labels above the bars indicate the absolute number of included articles.

Figure 2 shows the number of included articles per year of publication, categorically separated into non-COVID-19 and COVID-19 related research. We divided the number by the total number of articles published on PubMed for each respective year to account for the general growth in research output. Additionally, labels indicate the absolute number of included articles for each year.

We identified a clear upwards trend in papers published that utilize anonymized data over the years, which further intensifies with the onset of COVID-19. The regression analysis, conducted after normalizing against the annual total research registered on PubMed, reveals a statistically significant yearly increase of 2.16 included articles per 100,000 articles (*p* = 0.021). Even when excluding articles with COVID-19 focus, the upward trend amounts to an increase of 1.14 articles per year (*p* = 0.030).

### Geographical differences

**Fig. 3:**
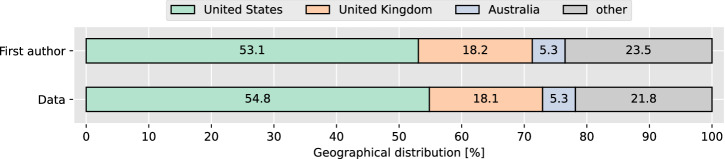
Geographical distribution of first authors and data provenance. Countries associated with the origin of less than 5% of first authors are categorized as “other”.

Figure 3 shows the distribution of the location of the first authors and the origin of the data sorted by frequency of occurrence for the papers where the data originated from exactly one country (1027 [94.7%] of 1084). In the great majority of these cases (970 [94.4%] of 1027), the location of the first author and the data origin was the same. The first authors came from a total of 53 different countries, while the data originated from 55 different countries.

Among the 1027 articles with data originating from a single country, the most frequent country of first authors was the US (545 [53.1%]), followed by the UK (187 [18.2%]), and Australia (54 [5.3%]). Similarly, for these articles, most data originated from the US (563 [54.8%]), followed by the UK (186 [18.1%]), and Australia (54 [5.3%]). Notably, these are all countries from the so-called Core Anglosphere, i.e. a group of English-speaking countries with historical, cultural, and political links, which includes the US, UK, Canada, Australia, and New Zealand. In contrast, only 10.1% (104 of 1027) of first authors and 8.7% (89 of 1027) of the data came from continental EU countries, Norway, or Switzerland, which all operate under the EU General Data Protection Regulation (GDPR) or a highly convergent law, and to which we henceforth refer to as Continental Europe. In the studies that sourced data from multiple countries (57 [5.2%] of 1084), the most common countries of first authors were the US (16 studies), the UK (11 studies), and Australia (5 studies), whereas the top countries from which the data originated were the US and UK (used in 13 studies), Germany (11 studies), Spain, France, and Italy (9 studies each). It should be noted that for 27 of the articles with multiple data origins it was not possible to break down the individual countries.

**Fig. 4:**
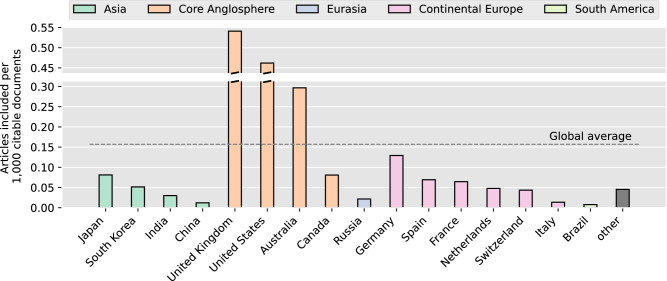
Number of articles per country. The number of articles with same author and data origin was normalized by the overall medical research output for each country, approximated by the number of citable documents in the field of medicine from 2018 to 2022, as indexed in SJR. Countries among the top-20 in research output assigned to at least one article are shown individually. All remaining countries were categorized as “other”.

Figure 4 displays the number of articles included per country, normalized by the number of citable documents of the country in the subject area of medicine (as a proxy for overall research output) in the years 2018–2022, according to the SCImago Journal and Country Rank (SJR)^36^. Countries to which we assigned articles and which are among the top-20 according to their medical research output in the SJR are shown individually (16 in total). All other countries listed in SJR are summarized in the category “other”, including 34 countries to which we also assigned papers. For an included article to be assigned to a country, both the first author and the data must be from that country, and from that country only.

In addition to the grouping of countries belonging to the Core Anglosphere, which was already identified as particularly relevant in the previous analysis, and Continental Europe, the categorization was extended with groups for countries within Asia, Eurasia, and South America. Eurasia was added to accommodate Russia’s unique geographic and geopolitical positioning across two continents.

For each group we calculated an average over all countries assigned to this group in the previous step. We did this by first summing up the number of articles assigned to those countries, and then dividing this total by the number of citable documents in the SJR for those countries. Countries from the Core Anglosphere, i.e. the UK, US, Australia, and Canada have by far the highest average ratio of articles included to the total number of publications (average of 0.345 articles per 1000 citable documents), exceeding the average of the top-20 countries shown in Fig. 4 (0.198) as well as the global average (0.157). Conversely, in Continental Europe, anonymized data sharing is comparatively uncommon, with such studies being underrepresented relative to the overall research output of these countries (average of 0.061). A similar picture can be observed for countries in Asia (average of 0.044). Notably, Germany and Japan rank fourth and fifth overall, with 0.129 and 0.081 articles per 1000 citable documents, respectively.

**Fig. 5:**
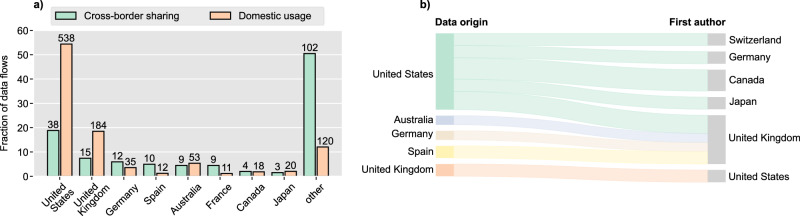
Patterns in cross-border data flows. **a** Proportion of data origins in cross-border and domestic data flows. Labels above the bars indicate the absolute number of data flows. Countries not listed at least twenty times as the origin of a data flow were categorized as “other”. **b** Sankey diagram of cross-border data flows found at least three times (39 data flows).

Next, we examined individual data flows by looking at the origin of the data and the first authors to understand how data has been transferred. We found that 10.5% (114 of 1084) of first authors have used data from one or multiple foreign countries, resulting in a total of 202 cross-border data flows. The great majority of authors (991 [91.4%] of 1084) used data from their own country either exclusively or in addition to foreign data, which we have counted as a total of 991 domestic data flows. Figure 5a shows the relative frequency of data origins in cross-border data flows as well as domestic data usages. Countries that are not listed at least twenty times as the origin of a data flow were categorized as “other”. Figure 5b illustrates specific cross-border data flows identified in at least three studies.

As can be seen in Fig. 5a, the US, which we found to be the country sharing data most often, was also the country sharing data most frequently in cross-border scenarios. Nevertheless, for both the US and the UK, cross-border data sharing was much less common than in domestic scenarios, both in absolute and proportional terms. This also explains why countries categorized under “other” have a comparatively large share of cross-border flows. Among these, there are 26 countries where we found only studies that used data in cross-border scenarios and none with domestic usage. The most frequent patterns shown in Fig. 5b reveal a notable predominance of cross-border data flows within the Core Anglosphere, as well as between the Core Anglosphere and Continental Europe.

### Common data sources

Of the 1084 studies included, 85.8% (930 of 1084) were identified through the use of healthcare related terms to describe their data, 8.4% (91 of 1084) used research data related terms, and 5.8% (63 of 1084) used both (see Table 3 for a list of corresponding terms). To gain a better understanding of the entities providing the data, we examined common sources, which we define as data sources utilized in at least ten studies. Table 1 summarizes these sources. More than 40% of the studies (460 [42.4%] of 1084) used data from one or multiple common sources. The share of articles utilizing common data sources has steadily increased over time, from 31.1% (44 of 140) in 2018 to 50.1% (167 of 330) in 2022. We also assessed whether repeated citations by author groups influenced a source’s classification as common by calculating the share of distinct authors among all citing authors. This was not the case: half (6 of 12) of the common sources exceeded 80%, and 83.3% (10 of 12) reached at least 70%. The only exceptions were SLaM NHS (59.3%) and VUMC (66.1%), which, however, had high citation counts (25 and 52), well above the 10-reference threshold. The distribution of the use of healthcare and research data terms in studies utilizing common data sources is comparable to that across the corpus.

**Table 1:** Common sources of data

Name	Country	Description
Cerner Corporation (Cerner)	US	Supplier of health information technology platforms acquired by Oracle in 2022. Offers access to longitudinal electronic health record data^59,67^.
Clinical Practice Research Datalink (CPRD)	UK	Funded by the Medicines and Healthcare products Regulatory Agency and the National Institute for Health and Care Research. Offers primary care data from general practices^60^.
Flatiron Health (Flatiron)	US	A healthcare technology company focusing on cancer care and research. Offers real-world cancer care data^68,69^.
IBM Watson Health (IBM)	US	A former division of IBM focusing on medical research and healthcare solutions acquired by Francisco Partners in 2022. Offers multiple datasets including IBM Explorys with routine healthcare data^70,71^.
Institute for Applied Health Research Berlin (InGef)	DE	Research institute connected to statutory health insurances through its owners. Provides anonymized claims data from multiple German health insurances^72^.
IQVIA	US	Global provider of health information and clinical research services. Offers real-world data, including electronic health records (EHR) and claims data^73^.
National Prescribing Service – MedicineWise (NPS)	AU	Funded by the Australian Government. Offers routinely collected health records to improve the surveillance of medicine use and primary care in Australia^61^.
Optum Incorporated (Optum)	US	A healthcare company. Offers various datasets including administrative data, claims data, and electronic health records^74^.
Secure Anonymised Information Linkage Databank (SAIL)	UK	Funded by the Welsh Government. Offers health and census datasets^46^.
South London and Maudsley NHS Foundation Trust (SLaM NHS)	UK	Funded by the UK’s National Health Service (NHS). Offers mental health data^47^.
TriNetX	US	A company focusing on real-world evidence generation by establishing a global network of healthcare organizations and life sciences companies. Offers access to longitudinal electronic health record and insurance data^75^.
Vanderbilt University Medical Center (VUMC)	US	Funded by Vanderbilt University. The Synthetic Derivative mirrors Vanderbilt’s electronic health record system and can be combined with samples from Vanderbilt’s BioVU biobank for genome-phenome analysis^39,76^.

Consistent with our previous findings, 58.3% (7 of 12) of the identified common sources are from the US. The vast majority of US common sources (6 [85.7%] of 7), as well as the German InGef are commercial enterprises. The only non-commercial US provider is the Vanderbilt University Medical Center, which predominantly supports intra-mural research. Common data sources located in the UK and Australia, are all publicly funded. All common sources offer at least some data that was routinely collected during healthcare processes. Except for TriNetX and IQVIA, for which some of the included articles indicate that data was gathered from multiple countries, the origin of the data used in the studies corresponded to the origin of the respective data source. We note that MIMIC, another well-known source of anonymized data^37^, was also used by some studies, but did not classify as a common source per our definition.

**Fig. 6:**
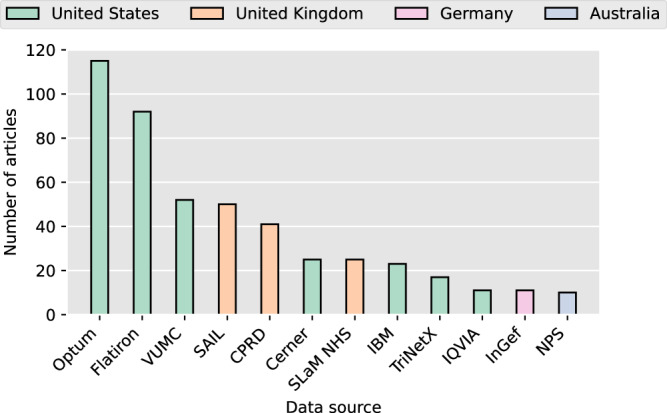
Common data sources. Each bar represents the number of studies in which a data source has been used.

Figure 6 provides an overview of how many articles used data from each common source. Due to some articles using multiple common data sources, the total number of usages amounts to 472. Notably, two healthcare and information technology companies, Optum and Flatiron, together account for 43.9% (207 of 472) of all uses of common data sources. VUMC stands out as the only common source operated by a single academic institution, ranking third in frequency of use and accounting for 11.0% (52 of 472) of all uses. It should be noted that 86.5% (45 of 52) of these uses involved intra-mural provisioning, where the first author was also affiliated with VUMC. Publicly funded entities in the UK cumulatively represent 24.6% (116 of 472) of the used common sources.

**Fig. 7:**
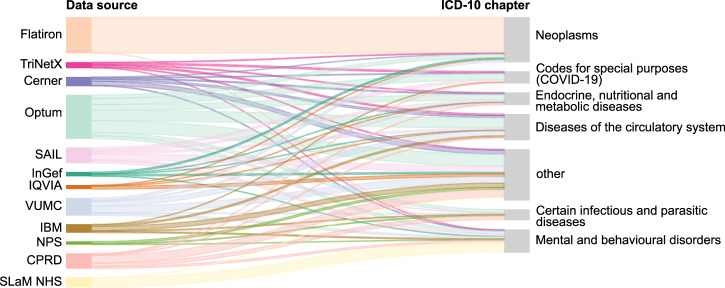
Flow of data between common sources and studies focusing on certain disease categories. ICD-10 chapters assigned to less than 5% of the articles included were classified as “other”.

Figure 7 illustrates the data flow from common sources to studies targeting specific disease categories. A substantial proportion of the studies on neoplasms use data from Flatiron (88 [51.8%] of 170), reflecting its specialization in cancer. Optum enabled a significant proportion of the studies on diseases of the circulatory system (14 [16.5%] of 85), while data from SLaM NHS was used often in studies on mental and behavioral disorders (24 [20.7%] of 116). Most of the common sources are not specialized but provide data for studies across a broad spectrum of diseases.

## Discussion

Our results show that the number of studies relying on anonymized data has steadily increased in recent years. The COVID-19 pandemic response, necessitating enhanced information flow, has further amplified this trend and the advancement of anonymization technologies is also called for in agendas for pandemic preparedness, such as the GLoPID-R Roadmap for Data Sharing in Public Health Emergencies^38^.

Regarding geographic, regulatory, and cultural differences, the sharing of anonymized data is particularly common in countries belonging to the Core Anglosphere. The US, UK, and Australia share anonymized data more frequently than other countries, even when put in relation to their overall research output. In contrast, countries in Asia, Continental Europe, Eurasia, and South America, seem to provide less anonymized data to biomedical studies. One reason for this are differences in legal and regulatory frameworks. In the US, anonymization is often performed based on the requirements laid out in the HIPAA Privacy Rule, for example in Vanderbilt’s Synthetic Derivative^39^ or MIMIC^37^, illustrating that the legal requirements are interpretable and can be applied consistently as a standard of practice. The GDPR, however, is interpreted differently across EU member states (and countries with similar laws, such as Switzerland, Japan, and Korea^40^) with ambiguous perceptions of anonymity^41^. It can be understood as an absolute state achieved solely by transforming the data^41,42^, or as a state that exists within a certain context^33^. As a result, despite a legal framework comparable to the GDPR, the sharing of anonymized data is much more common in the UK. In a report from 2006, the UK Academy of Medical Sciences noted that anonymity should be assessed within the context in which data is being shared^43^. This interpretation remained unchanged with the introduction of the GDPR in 2018^44^. Also in Australia, a context-sensitive approach is being pursued^45^. One common way to control the context is the sharing of data through secure platforms, called Safe Havens or Secure Processing Environments, which are for example used by SAIL^46^, SLaM^47^, and MIMIC^37^. This approach also forms a core concept of the upcoming European Health Data Space, which provides an example of a case where mechanisms and protocols successfully used in some countries have informed implementation strategies in others.

We acknowledge that further factors, such as technical, motivational, and economic barriers, influence data sharing practices, which may also impact anonymized data sharing^48^. However, there is ample evidence that data protection is a fundamental roadblock and that only once basic legal preconditions are sufficient, further aspects become critical factors. For example, a frequently cited barrier to data sharing is the lack of incentives, even within countries where anonymized data sharing is common, such as in the US^49–51^. At the same time, there are numerous large-scale data sharing initiatives in regions where we found anonymization to be less commonly adopted^52,53^. They are usually based on obtaining informed consent or on dedicated laws. TEHDAS, a collaborative initiative aimed at preparing and shaping the EHDS, also identified the lack of clear guidance on anonymization as a key barrier to data sharing^54^.

Our findings also showed that cross-border flows of anonymized data are relatively uncommon, even for countries which have a robust culture of domestic or intra-mural anonymized data sharing. This aligns with previous results. One explanation is that there are significant differences in data protection frameworks across jurisdictions, including the US, UK, and Australia, resulting in a lack of a uniform understanding^55^ and varying interpretations^56^. Moreover, it has been found that requirements for anonymization are often vague and that more comprehensive guidelines like the UK Anonymisation Decision-Making Framework^30^, which has also been adopted in Australia, are needed^55^. However, among recommendations on how to anonymize clinical trial dataset, a growing, yet incomplete, consensus on anonymization methods has been observed^57^. Recent publications from the International Standardization Organization (ISO) can be seen as a further step in the right direction^18,58^.

Finally, we observed that a significant portion of studies rely on anonymized data from common sources. These are usually focused on anonymized routine healthcare data, which is a natural application area for anonymization mechanisms. Many of the sources are located in the US and managed by commercial entities, alongside publicly funded entities in the UK and Australia. The only source identified outside the Core Anglosphere was InGef from Germany, a commercial provider with ties to statutory health insurances. The Vanderbilt University Medical Center was the only identified single academic institution that established a frequently used source of anonymized biomedical data. Some common sources as well as their data have also been described in the scientific literature^39,46,47,59–61^. Most common sources are not specialized but provide data to studies on a wide range of different diseases. Nevertheless, some identified sources focus on specific disease areas and contribute significantly to respective research using anonymized data.

We conclude that sharing and accessing anonymized data is an important mechanism enabling biomedical studies. Moreover, differences in regulatory frameworks and policies in different geographies have a measurable impact on how anonymization is adopted in practice. Our analysis focused on data protection-related differences and found that consistent policies and a context-sensitive interpretation of the concept of anonymity may help to implement anonymization on a broader scale. Moreover, a significant proportion of anonymized data sharing occurs through publicly funded as well as commercially operated common sources and their methodologies could serve as blueprints for developing approaches that also work in other regions. Finally, we found that cross-border data sharing is rare in practice, highlighting the need to establish uniform anonymization standards. Our overall recommendations for improving existing data protection policies and structures as well as developing future research agendas are summarized in Table 2.

**Table 2:** Recommendations

Improvement of policies and structures: Data protection policies should be centered on a context-sensitive understanding of anonymity. Moreover, there should be clear descriptions of how to evaluate whether data is anonymous in specific contexts. Ideally, policies should be uniform across different regions to enable cross-border data sharing. To achieve this, it can be beneficial to establish large entities specialized on anonymous data sharing. Recent standardization efforts and developments, like the EHDS, align with these recommendations.
Development of future research agendas: Research on data protection techniques should include studies on how the degree of anonymity can be robustly assessed. Moreover, research is needed that supports such assessments in specific contexts, where multiple protection methods are usually combined. This is crucial not only for anonymization methods but also for further privacy-enhancing technologies. Additionally, research is needed on techniques suited for data with very specific properties, such as data from patients with rare diseases, to improve the availability of anonymized data for further disease areas.

Limitations include a review scope restricted to studies indexed in PubMed, written in English, and published between 2018 and 2022, which could have introduced bias. Additionally, the interpretation and jurisdictional variations of the terms anonymization, de-identification, and pseudonymization might impact the generalizability of our findings. However, PubMed is one of the most comprehensive databases for biomedical literature and the selected timeframe is characterized by increasing privacy awareness and evolving regulatory landscapes (e.g., the introduction of the GDPR in the EU). We also carefully selected a terminology for our search criteria, and our findings confirm results of previous research conducted on the topic at a conceptual level. Our geographical analysis, based on the first author’s institution and data origin, may not fully capture global collaboration networks, though the rarity of cross-border data flows suggests limited impact on our conclusions. Moreover, while our study aims to provide a global overview, our findings, based on a subset of literature explicitly mentioning the use of anonymized data, might not be fully comprehensive. This can be exemplified by the absence of some widely recognized sources of anonymized data as common data sources, such as MIMIC^37^ and the UK Biobank^62^, possibly due to their prominence and policies affecting how studies reference anonymization. However, as our analysis mainly relies on the comparison of relative frequencies and trends, we believe that our results are representative. Ultimately, while we identified regional differences in the use of anonymized data that correspond with differences in data protection laws and their interpretation, there are further factors that also need to be considered when measures are planned to make anonymized data sharing more common.

## Methods

### Search strategy and selection criteria

We identified biomedical studies that produced findings based on the analysis of anonymized datasets published between 2018 and 2022 through searches of PubMed. This period includes the introduction of new data protection regulations in several countries and regions, such as in Brazil, China, the EU, Japan, and Thailand, the peak of the COVID-19 pandemic (2020-2021) and the time thereafter. Where applicable, we reported our review process in accordance with the PRISMA checklist for systematic reviews^63^, and employed a step-wise procedure for fine-tuning our search query.

Selecting studies that utilized anonymized biomedical data published by authors across the globe is complicated by the varying terminologies used in different regions and contexts^56^. The terms anonymization and pseudonymization are, for example, used in countries within the EU, the UK, and China. We deliberately focused on anonymization and not on pseudonymization (sometimes also referred to as pseudo-anonymization^18^), as it is generally understood that pseudonymized data retains a protected but explicit link back to the individual. Despite debates on what exactly distinguishes pseudonymized from anonymized data, the latter is commonly understood as data that cannot be traced back to individuals. This aligns with the common understanding of the term de-identified data^56^, for example in the US, Canada, or Australia. For instance, HIPAA defines de-identified health information as data for which there is no reasonable basis to assume it can be linked back to an individual^64^. In contrast, pseudonymization is usually used during data collection or when patients have explicitly consented to the use or sharing of their data^65^.

Our initial search utilized terms related to anonymization and de-identification along with their spelling variants, combined with keywords pertaining to various types of healthcare or research data. We refined our search by excluding terms associated with privacy, surveys, and legal issues to focus specifically on studies that use anonymized health data rather than addressing the topic on a meta-level. All search terms were matched against titles and abstracts. The individual groups of terms as well as the final search string is shown in Table 3. The final search was performed on 06.02.2025.

**Table 3:** Search string

Search concept	Search terms
Anonymization terms	“anonymization” [Tiab] or “anonymisation” [Tiab] or “de-identification” [Tiab] or “deidentification” [Tiab] or “anonymized” [Tiab] or “anonymised” [Tiab] or “de-identified” [Tiab] or “deidentified” [Tiab]
Healthcare data terms	“health data*“[Tiab] or “clinical data*“[Tiab] or “health record*“[Tiab] or “routine data*“[Tiab] or “medical data*“[Tiab] or “medical record*“[Tiab] or “healthcare data*”[Tiab] or “patient data*”[Tiab] or “EHR”[Tiab] or “EMR”[Tiab]
Research data terms	“trial data*“[Tiab] or “study data*“[Tiab] or “participant data*“[Tiab] or “IPD”[Tiab] or “research data*”[Tiab] or “subject data*”[Tiab] or “patient-level data*”[Tiab] or “participant-level data*”[Tiab] or “subject-level data*”[Tiab] or “biomedical data*“[Tiab]
Exclusion terms	“questionnaire*“[Tiab] or “interview*“[Tiab] or “ethic*“[Tiab] or “legal*“[Tiab] or “privacy*“[Tiab]
Final query structure	(Anonymization terms) and (Healthcare data terms or Research data terms) not (Exclusion terms) Restricted to publication years 2018 to 2022

During the screening process, we excluded studies (1) focusing on anonymization technology, (2) which did not present findings that were produced using anonymized data, (3) which did not focus on a health-related outcome, (4) which were solely based on hard-to-anonymize unstructured data, (5) were based on anonymous surveys, or (6) focused on the implementation of data sharing. The screening process involved an initial screening of titles and abstracts by two reviewers assigned at random. Discrepancies in their decisions were resolved through discussion with a third reviewer. Subsequently, full-text articles were evaluated for inclusion using the same procedure.

### Data collection and analysis

Table 4 lists the data items we collected to provide insights into the three dimensions of interest. To investigate temporal trends, we charted the year of publication as well as whether a study produced COVID-19-related findings, to see whether the pandemic response had an effect. To provide insights into geographical differences, we collected the country in which the first author’s first affiliation is located as well as the country of origin of the dataset used in the study. We defined the dataset’s origin as the country in which the data was initially collected, typically corresponding to the location of the data source (e.g., hospitals, insurance companies, or research institutions) providing the data. In cases where data was obtained from multiple countries, all contributing countries were recorded, provided they were reported separately. We note that we also collected information on the senior author’s locations, but those were largely consistent with the origin of the first author (same in 1014 [93.5%] of 1084 cases). Finally, to study common data sources, we charted whether a paper used terms related to healthcare or research data or both (see Table 3), the name of the source providing the data, as well as the ICD-10^66^ chapter of the diseases of interest. Analogously to the data origin, several entities could be recorded as data sources for a single article. When a study spanned morbidities from multiple ICD-10 chapters, the journal’s subject area was utilized to identify a primary chapter. With the exception of the items “Year” and “Data type”, which were automatically inferred by querying the database, the charting was performed manually. Each item was charted by two independent reviewers and discrepancies were resolved by discussion with a third reviewer.

**Table 4:** Collected data items

Item	Description	Example	Dimension
Year	Year of publication	2020	Temporal trends
COVID-19 research	Whether the paper produced COVID-19-related findings	Yes	
Location of first author	Country of the institution listed as the first affiliation of the first author	UK, US	Geographical differences
Origin of data	Country or countries in which the data was collected	UK, US	
Data type	Whether the paper was included through the use of terms related to healthcare data, research data, or both	Healthcare data	Common data sources
Data source	Entity or entities stated as the source of the data	Optum, Flatiron	
ICD-10 chapter	ICD-10 chapter of the diseases studied	1, 2, 3	

According to our dimensions of interest, we prepare the results using primarily descriptive statistics, complemented by data flow mappings and clustering of data sources. Additionally, we supplement our data with external information on the general research output of countries using the SCImago Journal and Country Rank^36^.

## Data Availability

All data collected for this review is publicly available on GitHub (https://github.com/bih-mi/anonymization-review/).

## Code availability

The data analysis was performed using Python 3.11. The code is publicly available on GitHub (https://github.com/bih-mi/anonymization-review/).
